# A qualitative study on midwives’ identity and perspectives on the occurrence of disrespect and abuse in Maputo city

**DOI:** 10.1186/s12884-020-03320-0

**Published:** 2020-10-19

**Authors:** Anna Galle, Helma Manaharlal, Sally Griffin, Nafissa Osman, Kristien Roelens, Olivier Degomme

**Affiliations:** 1 International Centre for Reproductive Health, Department of Public Health and Primary Care, Ghent University, Corneel Heymanslaan 10, entrance 75, UZP 114, 9000 Ghent, Belgium; 2 International Centre for Reproductive Health, Rua das Flores no 34, Impasse 1085/87, Maputo, Mozambique; 3 Faculty of Medicine, Department of Obstetrics/Gynecology, Eduardo Mondlane University, Av. Salvador Allende 57, Maputo, Mozambique

## Abstract

**Background:**

Midwifery care plays a vital role in the reduction of preventable maternal and newborn mortality and morbidity. There is a growing concern about the quality of care during facility based childbirth and the occurrence of disrespect and abuse (D&A) worldwide. While several studies have reported a high prevalence of D&A, evidence about the drivers of D&A is scarce. This study aims to explore midwives’ professional identity and perspectives on the occurrence of D&A in urban Mozambique.

**Methods:**

A qualitative study took place in the central hospital of Maputo, Mozambique. Nine focus group discussions with midwives were conducted, interviewing 54 midwives. RQDA software was used for analysing the data by open coding and thematic analysis from a grounded theory perspective.

**Results:**

Midwives felt proud of their profession but felt they were disrespected by the institution and wider society because of their inferior status compared to doctors. Furthermore, they felt blamed for poor health outcomes. The occurrence of D&A seemed more likely in emergency situations but midwives tended to blame this on women being “uncooperative”. The involvement of birth companions was a protective factor against D&A together with supervision.

**Conclusion:**

In order to improve quality of care and reduce the occurrence of D&A midwives will need to be treated with more respect within the health system. Furthermore, they should be trained in handling obstetric emergency situations with respect and dignity for the patient. Systematic and constructive supervision might be another promising strategy for preventing D&A.

## Background

Midwifery care has an essential role in the reduction of preventable maternal and newborn mortality and morbidity worldwide [1]. Over the last two decades, there have been calls to prioritize the intra-partum period and promote facility delivery to improve maternal and newborn health outcomes [1]. As a result, more women are delivering in a health facility with a skilled birth attendant [2, 3]. However, there is a growing concern about the quality of the care that women are experiencing inside health facilities and reports of disrespectful and abusive treatment during labour and delivery continue to appear in many parts of the world [4–6]. In light of these concerns, the World Health Organisation (WHO) published a new framework for maternal and newborn health in 2016, which included an increased focus on respect and preservation of dignity [7]. Based on this framework, experience of care is an essential element of quality of care, which requires competent and motivated human resources as well as the availability of essential physical resources [8]. According to WHO, health systems must be accountable for the treatment of women during childbirth, ensuring clear policies on rights and ethical standards.

Mozambique has a long history of civil war which compromised the development of a functioning health system, but since the signing of a peace agreement in 1992 the country has made significant progress in providing health for all [9]. Nevertheless, maternal and newborn health outcomes are still among the worst in Sub Saharan Africa. The most recent estimates report a maternal mortality ratio of 289 maternal deaths per 100 000 livebirths in 2017 [10] and only 54% of births attended by a skilled birth attendant in 2015 [11]. Several actions have been taken to improve maternal and newborn health in the last decade. After years of investing in scaling up the number of health care providers and health facilities [12], in 2007 the Ministry of Health (MoH) has made humanization and patient friendly care during antenatal care (ANC) and delivery one of its priorities, recognizing the importance of quality of care. Over time, the culture of promoting Respectful Maternity Care (RMC) has become more widespread in Mozambique and the MoH has transformed a selection of maternity wards into centres of quality and humanized Maternal and Newborn Health (MNH) care provision under the “Iniciativa Maternidade Modelo” (Model Maternity Initiative). The limited evidence shows most women (92%) in Southern Mozambique are satisfied about the interaction with the health care provider in maternity care and that the prevalence of disrespect and abuse (D&A) varies among settings and regions; from 27% in the referral hospital up to 70% in more rural facilities [13, 14]. The WHO defines the occurrence of D&A in childbirth as interactions or facility conditions that local consensus deems to be humiliating or undignified, and those interactions or conditions that are experienced as or intended to be humiliating or undignified [4].

Midwives are the key frontline health workers in providing maternal and newborn health care in Sub-Saharan Africa, operating in rural and urban areas in often challenging health systems. While most Low and Middle Income Countries (LMIC) have a well-defined rural health system, with a focus on primary care and often extensive cadres of community health workers and volunteers, the same structures rarely exist in cities [15]. Focusing on Maputo, the capital of Mozambique, the health infrastructure consists mainly of public facilities, some supported by non-governmental organizations (NGOs), and a smaller but increasing number of private clinics [16]. The city is facing a brain drain of health care workers from the public system to the private sector (including private clinics, development agencies and NGOs), where salaries and working conditions are much better [16, 17]. Continuity of care and functioning referral systems are a major challenge and appropriate gatekeeping to limit the number of patients using tertiary care who could be better served in primary care is limited. As a consequence, the daily challenges faced by midwives working in cities are likely to be different to those in rural areas.

Various cross sectional studies have explored the occurrence of D&A, listening to women’s voices, both quantitatively and qualitatively [5, 14, 18, 19]. However, for designing effective prevention programs the drivers of D&A need to be explored together with the working environment in which disrespect occurs [20]. The limited literature on midwives’ perspectives regarding D&A in maternity care indicates that organizational difficulties, lack of accountability and an ideology of patient inferiority are frequently cited causes of D&A [21–23] . Taking into account the increasing urbanization and modernization of most African cities, there is a need to explore the specific challenges midwives might face in urban public facilities and potential causes of D&A in these settings. Causes of D&A in urban settings may differ from those in rural areas as there is generally a more varied patient population, higher availability of doctors and provision of private care within and outside of the public hospital. With this study we aim to explore midwives’ professional identity and perspectives on the occurrence of D&A in urban Mozambique.

### Setting

Mozambique has a general shortage of health care providers but the MoH is gradually scaling up the number of health care workers as well as their professional training and availability and accessibility of postgraduate courses [24, 25]. The standardized national curriculum of midwifery education requires 4 years of studying, followed by continuous professional development through in-service training and refresher courses [24]. The study was conducted in Hospital Central de Maputo (HCM) in Mozambique’s capital city. HCM is a tertiary referral hospital with on average 20 deliveries a day. On the delivery ward four midwives work each shift, together with one senior obstetrician and one junior resident. A full-time position as a midwife constitutes of 40 working hours. It is noteworthy that midwives in Maputo City often combine a job in the public sector with extra hours in the private sector to increase their income. HCM is the only hospital in the country equipped to handle advanced operations, thereby serving as the last referral centre for the entire country [26]. The principle investigator (AG) has been leading a cross sectional study about RMC in the same hospital [14] and was involved in various projects in the hospital between 2014 and 2019, witnessing the evolutions in terms of equipment, infrastructure and quality of care over this period. The maternity ward has improved substantially between 2011 and 2018, through expansion of infrastructure and strengthened quality standards. While prior to 2016 there was just one delivery room with all women delivering side by side, all women in active labour now have separate rooms. Despite the scale up in terms of infrastructure, essential medicines and equipment are still scarce, and are stored centrally in the corridor. Over this period the hospital has transformed into a center of quality and humanized MNH care. Respectful maternity care is one of the essential packages of the model and includes respect for beliefs, traditions, and culture; the right to information and privacy; choice of a birth companion; freedom of movement and position; skin-to-skin contact and early breastfeeding; appropriate use of technology and effective lifesaving interventions; and prevention of violence and disrespectful care [12].

### Research team

The principal investigator (AG) is a Belgian doctoral student with a midwifery degree and research experience in Mozambique. A final year medical student (HM) assisted during all focus group discussions (FGDs) with note taking and guiding the discussions.

### Participants & study procedures

Data collection took place between May and June 2019. FGDs were chosen as the data collection method because we wanted to create a dynamic discussion about the professional identity of midwives and get different perspectives on D&A [27]. All midwives of the central hospital involved in obstetrical care were invited to be interviewed as well as the head midwives. FGDs took place with midwives of the delivery ward and the maternity ward, the majority of the midwives rotate among the wards and all are involved in obstetrical care. FGDs were conducted in a private meeting room in the hospital and took place at the end of midwives’ shifts. In total 56 midwives were invited for the focus group discussions, of which two refused due to unavailability at the time of the interview. Head midwives were interviewed in separate FGDs to allow for openness among participants. All FGDs were facilitated in Portuguese by the researcher (AG), assisted by a local research assistant (HM). In the first part of the discussion midwives were asked how they felt about their profession, their role in the hospital and in society. The second part of the discussion focused on exploration of their understanding of respectful maternity care and the main reasons for the occurrence of disrespect and abuse during labour and delivery. The interview guide can be found as an additional file (see additional file 1).

### Data analysis

**Fig. 1:**
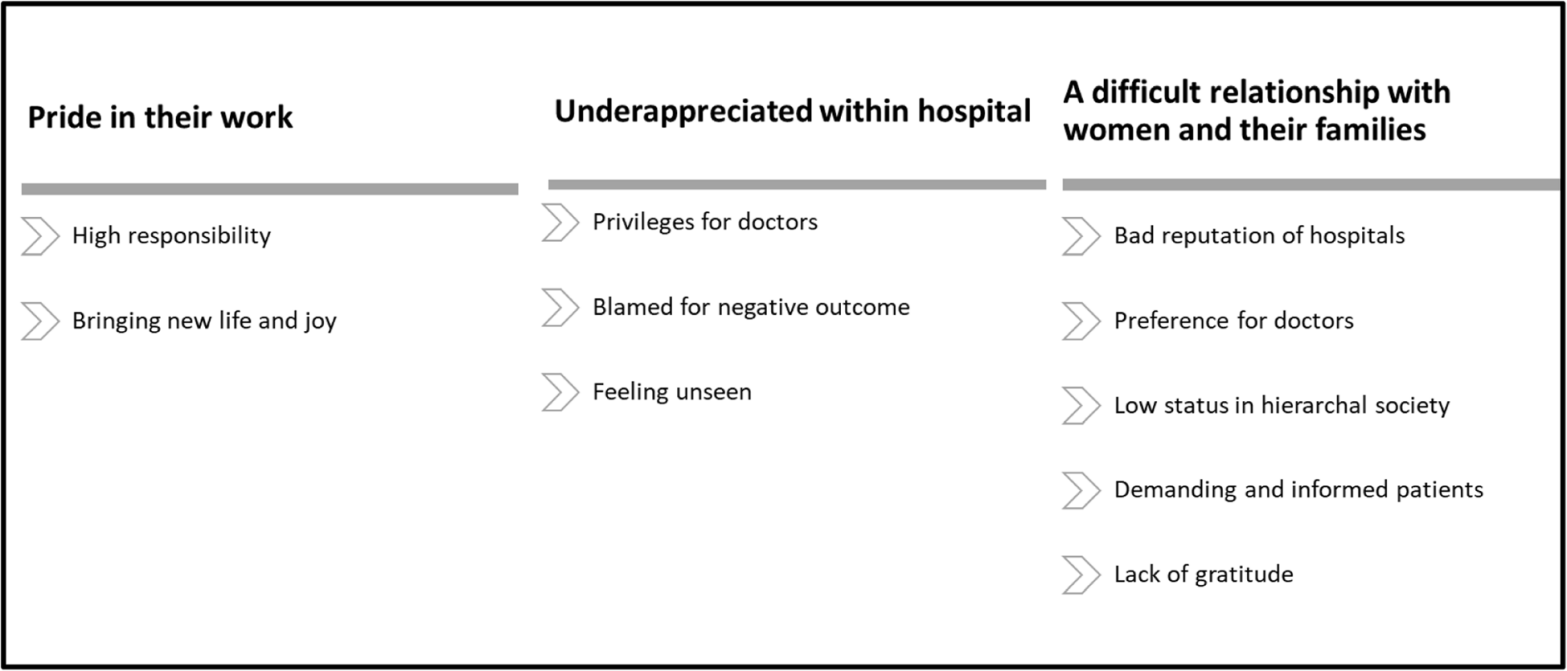
Midwives’ identity

**Fig. 2:**
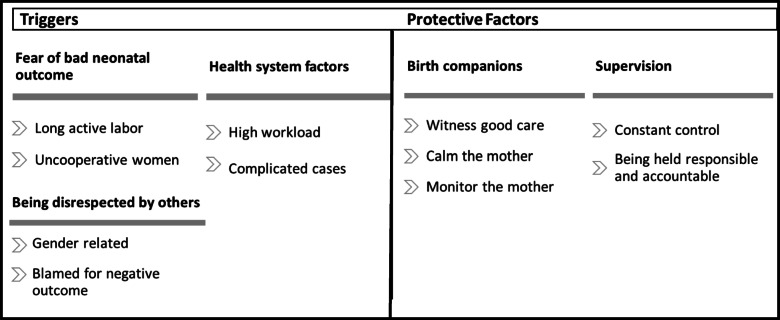
Triggers and protective factors of D&A

All focus group discussions were transcribed verbatim in Portuguese by HM and were double-checked by AG. Braun & Clarke’s six-phase framework was used during thematic analysis and open coding [28, 29] was applied. This framework involves a reflexive process of moving forwards (and sometimes backwards) through data familiarization, coding, theme development, revision, naming, and writing up [29, 30]. A grounded theory approach was used for the identification and progressive refinement of important themes from the data [31]. The final themes can be found in Fig. 1 and Fig. 2.

R Qualitative Data Analysis (RQDA) software, an R package, was used for coding. All data were coded by both AG and HM, all codes were discussed together after each FGD and divided into themes. All analyses were carried out in Portuguese. Only age ranges were reported along with quotations to guarantee anonymity.

### Ethical considerations

The health directors and head midwives were contacted for authorization and assistance in organizing the FGDs. Information about the objective of the study and procedures was provided to all respondents verbally and in writing. Participants were asked if they consented to interviews being recorded using a tape recorder. Confidentiality, anonymity and ground rules were discussed before starting the FGD. Participation in the study was voluntary and all participants gave their written consent. No incentive was provided, other than refreshments during FGDs. Ethical approval for the study was obtained from the medical ethical commission of Ghent University (EC/2018/1319) and the Health Bioethics Committee of Universidade Eduardo Mondlane (UEM) and HCM (CIBS UEM&HCM/0008–17).

## Results

In total 54 midwives participated in nine different FGDs (see Table 1). The number of participants per focus group ranged from five to seven. During analysis we identified two main themes: midwives’ identity (summarized in Fig. 1), and factors affecting the occurrence of disrespect and abuse (see Fig. 2). While we report these themes separately for clarity, the two themes were clearly related to each other and sub themes often overlapped. In particular, the subthemes “underappreciated within hospital” and “being disrespected by others” intertwined and were part of the two core themes “midwives identity” and “the drivers of D&A” respectively. The coding structure and frequency of occurrence of each code in each interview can be found as additional files (additional files 2 and 3, respectively). The sociodemographic characteristics of the participants are shown in Table 1, and emerging themes and sub codes are discussed below.

**Table 1:** Socio demographic characteristics of participants

**Sociodemographic characteristics**	Participants % (n)
• 24–30	26.79 (15)
• 31–40	42.86 (24)
• 41–50	14.29 (8)
• 51–61	16.07 (9)
**CHILDREN**	
• Yes	83.93 (47)
• No	16.07 (9)
**RELIGION**	
• Catholicism	46.30 (25)
• Islam	1.85 (1)
• Cristian	31.48 (17)
• Others	20.37 (11)
**POSITION WITHIN TEAM**	
• HEAD MIDWIFE	22.22 (12)
• MIDWIFE	77.78 (42)
**TOTAL NUMBER OF PARTICIPANTS**	54

### Midwives’ professional identity and role in society

#### Pride in their work

Midwives all were proud of their work. They are involved in the process of bringing new life, which is a high responsibility and brings a lot of joy. This was described as follows:

*“Being a midwife is not just a job, we are actually helping people. And we have to do it with our heart, that is most important.”* (Midwife, FGD 5, age group 41–50).

Most women are also satisfied and happy after the delivery, and especially at the maternity ward the contact with women was told to be positive.

*“Most women got what they came for. So they are happy.”* (Midwife, FGD 6, age group 51–60).

Midwives reported being respected by their family and wider community, especially when they grew up in rural areas, where they were often regarded as educated and “medical doctors”.

Gratitude by patients brought midwives satisfaction in their work and gave them motivation to overcome all challenges and difficulties.

*“As a midwife you are responsible for two lives, which is a huge responsibility, so you want to do it with perfection.”* (Midwife, FGD 7, age group 31–40).

However, they also revealed that in the city they are losing this unanimous appreciation and linked this to broader access to information and services.

*“Since they can look up everything on internet they believe they know better than us, they come and say I want this and this. They don’t show respect anymore.”* (Midwife FGD 1, age group 31–40).

#### Underappreciated within the hospital

Midwives disclosed that their work and efforts were not always appreciated within the hospital, especially compared to the appreciation and privileges that doctors received.

*“Respect has to be mutual. I respect you, you respect me. If there is some kind of disrespect between the two the other one will not feel comfortable. And in this hospital, in this institution, midwives are not respected.”* (Midwife, FGD 2, age group 31–40).

Doctors were treated better at the hospital by receiving small benefits such as better food at lunch or having cold water at their disposal. Other benefits exclusively for doctors mentioned included direct access to hospital services for relatives. Midwives perceived this preferential treatment as wrong. Younger midwives seemed to be especially bothered by the unequal treatment compared to doctors.

*“Even our own hospital discriminates between doctors and midwives. Their room for refreshment is much better equipped, they always have water and also the food is much better than what we get.”*(Midwife, FGD 5, age group 31–40).

However, it is important to mention that all midwives stated they felt respected by doctors in their direct working relationship. In the delivery room their opinions were heard and collaboration was mostly productive and with respect for each other. The problem was rather an institutional discrimination between the two professions. A strong bond among colleagues was one of the most important enablers for midwives to fulfill their job with positivity and satisfaction.

*“For me a good day at work means you enter, say good morning and can talk and joke with your colleagues in a good atmosphere.”* (Midwife, FGD 2, age group 24–30).

The hospital carries out security checks at the gate for everyone who enters or leaves the hospital, with no distinction made between patients, visitors or personnel. Medical personnel are often searched at the gate, which midwives perceive as very disrespectful and humiliating.

*“At the end of the day we are tired and want to go home, but at the gate we are being searched by security guards, in front of our own patients. Just like we are thieves. That is humiliating.”* (Midwife, FGD 9, age group 24–30).

Midwives felt discriminated and targeted during audits for medical errors. Although doctors were also questioned during audits, midwives felt they were often held responsible for errors because they look after the patient, which they explained was a constant stress. They also perceived as wrong the fact that they are never informed of the results after an autopsy of a maternal death, while doctors are always informed. These events affect the team spirit in a negative way.

*“When a medical error is found they will always point at us. Just because we are the lowest rank in the hospital. That is how it is.”* (Midwife, FGD 1, age group 31–40).

#### A difficult relationship with women and their families

A serious challenge in midwives’ relationship with women and their families was linked to the poor reputation of public hospitals. The idea that many patients die *because of the hospital* (and not because of their health problem) is very prevalent in society.

“*Most patients don’t appreciate our work. They blame us for all their bad experiences with hospitals, it is all our fault.”* (Midwife, FGD 7, age group 24–30).

Insults and aggression by patients was a daily reality according to the midwives, mostly by upper-class patients who demanded a better service. In addition the midwives explained they often experienced aggression by women who were not able to cope with the pain (for example, slapping or scratching the hands of midwives during painful procedures). Midwives stated the hospital management did not recognize these challenges or offer any assistance. A big frustration was that patients can easily lodge complaints (in complaint boxes) but that nothing is in place for reporting problematic behaviour of patients towards health personnel.

The low status of midwives compared to doctors was also reflected in patients’ behaviour. As doctors are available in the tertiary hospital, some patients prefer their opinion and even refuse to accept midwives as their carers during normal labour and delivery. Women with a high status in society in particular tend to disrespect the profession of midwives.

*“Only by the time the woman has completed dilatation the doctor comes in and does the delivery. But I was following up that woman the whole day. Guess who they will thank? The doctor.”* (Midwife, FGD 7, age group 31–40).

Midwives mentioned they often felt treated as “servants” of the women. The existence of a private system in the public hospital tends to aggravate the problem. These patients expect a better service but they are treated in the same public hospital by the same health care providers (with limited time). In reality they only have a better equipped room which does not always meets their expectations.

“*These private patients expect me to sit next to them and do everything, they don’t want to get out of the bed. But I have 20 other women on the ward so I can only give her the same as all the others.”* (Midwife, FGD 6, age group 51–60).

### Occurrence of disrespect and abuse

### Triggers

#### Health system factors

Midwives mentioned that the lack of personnel is one of the major causes of why women are abandoned during labor and/or delivery. This is most problematic in rural health centers (where one nurse/midwife is often responsible for postnatal care, antenatal are, family planning and deliveries), but there are also some challenges associated with workload in the central hospital. Although it is a referral centre, there are not strict admission criteria which results in a very mixed patient population and high influx of patients. Midwives declared that they sometimes felt overwhelmed by complicated cases, especially during night shifts, which increased the risk of neglect.

*“If you are dealing with three patients and one has eclampsia, another needs a caesarean section and the third suddenly has a haemorrhage, for sure one will be abandoned. “*(Midwife, FGD 2, age group 24–30).

They also linked this to the stress of being accused afterwards of making medical errors.

*“When we have a lot of patients we have stress. But when we have mother that is not good we have a different stress, a psychological stress. Because we know she might end up dying on our ward, in our hands.”* (Midwife, FGD 6, age group 41–50).

#### Being disrespected by others

Midwives explained that the disrespect they receive from others will affect their relationship with the patient. During rounds in the mornings they were often blamed for mistakes.

*“You have to start the day and they [management/superiors/peers in the hospital] already insulted you. And this will affect your work with the patients, because your head is not there, it’s full already. They stressed you so basically your day is ruined already. And you will put your frustrations on the patient, it is the patient who will pick up the bill.”* (Midwife, FGD 3, age group 31–40).

Gender-related disrespect by patients was also mentioned by one midwife.

*“They might just slap or scratch you when you are working. You think they would do the same to a man? I don’t think so. It is just because we are women.”* (Midwife, FGD 1, age group 31–40).

They also mentioned problems with visitors who did not want to respect the visiting hours on the maternity ward. It was not unusual for midwives to have to call the security guards for assistance.

#### Fear of bad neonatal outcome

At critical points such as expulsion, midwives wanted to minimize the time and maximize their control over the situation in order to guarantee a good outcome.

*“If we are yelling at the mother it’s mostly for the interest of the baby. And the mother will even thank us for that afterwards.”* (Midwife, FGD 1, age group 24–30).

If the expulsion phase is taking too long they were convinced that it is necessary and acceptable to use force. Surprisingly, midwives unanimously tend to blame the women for a difficult delivery. They explained this might happen because women do not “collaborate”, or are too young or unexperienced.

*“The women that say we slap them or yell, are the ones that don’t collaborate. Even yesterday a mother was closing her legs and I lost control because the baby was suffering. I yelled at her: did you carry a baby for nine months to end up here closing your legs?”* (Midwife, FGD 3, age group 31–40).

### Protective factors

#### Birth companions

Midwives highlighted the benefits of allowing birth companions, for both the midwife and the patient. They can calm and reassure the pregnant women during labour, check up on the mother, help with small tasks and also witness good care. They were convinced that this might improve the reputation of the hospital.

*“Bringing in birth companions was a good thing, they are seeing everything we do. They can see we are not beating the women. I hope they also tell that to the other mothers.”* (Midwife, FGD 8, age group 51–60).

They also explained that even when they use force or yell, the birth companions can witness that they had no choice and were providing the best care possible. Most midwives were also in favour of inviting male birth companions. They explained that some women are asking to allow their husbands on the ward, especially because this is already happening in many private facilities. However, some midwives explained that an unprepared man might also be traumatized or uncomfortable in the delivery room. Therefore, they proposed two main precautions before allowing men: preparation of the husband during ANC and introduction of stricter privacy measures (currently the doors of all rooms are always open to facilitate monitoring of women).

*“Some women ask for their husband. But we cannot let them enter because we only have one corridor. Women walk half-naked and have contractions in the corridor. A man cannot see all that.”* (Midwife, FGD 7, age group (51–60).

#### Supervision and control

Although midwives clearly stated that the feeling of being controlled and checked all the time was a source of stress, they were convinced that this was one of the major reasons why the occurrence of D&A was relatively low in the central hospital. This in contrast to the districts where they described some level of immunity from punishment.

*“That one in the district can just do what she wants. We have our head midwife correcting us on the spot”.* (Midwife, FGD 8, age group 41–50).

All midwives seem to respect their head midwife. Head midwives in the hospital are chosen by a voting system among midwives. Midwives appreciated this system because a higher medical degree does not automatically give someone a higher position. Midwives with good interpersonal skills and experience were most often elected. Besides supervision and control by colleagues and superiors also a complaint system for patients was in place (by means of complaint boxes in the hospital to report improper care).

## Discussion

### Being a midwife in a national referral hospital

Our study started by exploring the meaning of being a midwife in an urban referral hospital. We tried to capture how midwives felt about their profession and their social identity in society [32]. To start on a positive note, pride and awareness of their high responsibility in taking care of mother and baby were frequently emerging themes. This commitment and empathy was also described by Adolphoson et al. in 2016, interviewing midwives in different settings in Mozambique [33]. On the downside, the recent evolution of having more demanding and informed patients together with a parallel private system are factors putting pressure on midwives working in the public system.

Globally, midwifery is commonly described as highly emotional and challenging work, with midwives experiencing many work-related conflicts and medical dilemmas [34]. While the relationship of midwives with doctors was generally good in our study, criticism and blaming by other colleagues (including midwives, doctors and superiors) eroded their morale. Other issues hampering job satisfaction were a lack of patients’ respect and lack of institutional recognition and support for their work. Professional empowerment of midwives could be a useful strategy to increase job satisfaction and quality of care [35, 36], but specific evidence for implementing interventions in a Mozambican context is lacking. The “Perceptions of Empowerment in Midwifery Scale”, a survey that has been implemented in various countries, could be a useful instrument to get more insight into the specific workplace factors affecting midwives empowerment in Mozambique [37–39] in order to inform the development of appropriate interventions.

Our study showed that the private health system clearly has an influence on the public system, for example patients expect higher standards of care, and some practices from the private system might influence the public system. However, current research and funding opportunities tend to focus on the public system only, resulting in limited evidence about parallel private systems and the interaction with public health systems. Greater emphasis and research on the influence of the private medical sector on the public sector is highly recommended, especially in these rapidly changing urban environments where the private health care system is growing [15]. Establishing effective public-private partnerships could be a way forward to improve quality of care, provided that they also guarantee universal access to health care [40].

Our study revealed that midwives are often blamed for negative health outcomes and are insulted within the hospital. Furthermore, the better working conditions for doctors were felt to be deeply unfair. Disrespect for midwives seems to be a global problem, WHO reported in 2016 that midwives often face discrimination, harassment and lack of respect worldwide [41]. Furthermore the WHO study showed that these negative experiences hinder midwives in their ability to provide quality care to women and newborns [41]. This was confirmed in our study, with midwives reporting that being insulted or disrespected by superiors at the start of their shift negatively affected their interactions with the patients for the rest of the day. Some authors have suggested that health care providers abuse patients to create a social distance and maintain identity and power in their continuous struggle to assert their professional and middle class identity in society [23]. While we lack evidence to apply this theory to the Mozambican setting, we can say that midwives in our study clearly struggled with their position in the institution and wider society. A clear non-discriminatory institutional policy and (peer) support system for health care providers could help increase job satisfaction for midwives and allow sustainable quality improvement of maternity care [42, 43]. Strengthening the national midwifery association could be a way forward to advocate for midwives’ rights.

### Enabling and protective factors of disrespect and abuse

After exploring the professional identity of midwives we explored their views on the occurrence of D&A in maternity care. We consistently use the terms “disrespect and abuse” in our study as defined by WHO [4]. Although other authors sometimes use the term “obstetric violence”, we found in a previous study that the most common forms of D&A in the Mozambican context do not align with theories regarding violence or aggression [14]. According to WHO’s definition [44], violence is always performed with the intent to harm. Our study showed midwives most important reason to use “obstetric violence” or conduct D&A was to save the baby’s life, which is in line with other studies [22, 45]. We can argue they could and should use other techniques to save the baby, but still their primary intention is not to hurt the mother. Therefore, we purposely never used “obstetric violence” in our study and believe this term should be used with caution in the literature. Referring to obstetric violence within this context might compound midwives’ feelings of being disrespected and blamed.

Relying on midwives’ previous experiences working in other settings, we were able gain insight in factors affecting D&A in different settings. In our study midwives reported that the main reason for a higher occurrence of D&A in rural areas in Maputo Province is the lack of supervision and accountability in these working environments. As supported by the literature, strengthened supervision will be a way forward to prevent the occurrence of D&A and improve the quality of care [46, 47]. Some promising results have been achieved in other settings by establishing peer support and supervision groups to reduce stress and increase professional skills [42, 43, 48]. The election of head midwives within the team was found to be a positive element of supervision in our study and could be a promising strategy for establishing non-punitive supervision in other health institutions.

The occurrence of serious emergency situations and a high workload seem to be risk factors for the occurrence of D&A in our study. Especially when the midwife fears for the baby’s health, she might use force to speed up the delivery (for example with fundal pressure during second stage of labor). Midwives in LMICs are not always trained and equipped to closely monitor fetal health, which increases uncertainty about the fetal condition and probability to intervene aggressively. In-depth counselling with the women could make certain interventions less traumatic, but providers in LMICs also lack training and time to invest in counselling [49]. Furthermore, proper pain relief for women (such as epidural analgesia) is often absent. Despite numerous studies examining D&A, an association between serious emergency situations and the occurrence of D&A has been little explored. In line with the work of Afulani et al. [22], the narratives of our respondents show that stressful situations and not feeling capable to manage these situations are triggers for D&A. Furthermore, midwives tend to blame women for a difficult labour by reasoning they are too young or not collaborating. Further studies should look more into ways to avoid D&A during specific emergency situations such as foetal stress or obstructed labour. Furthermore, midwives’ educational curriculum should include proper training about (pain) mechanisms during labour for avoiding such negative reasoning that might constitute to D&A. Some promising results have been found from the implementation of a workshop called “Health Workers for Change” in Tanzania covering reflection and discussion about different topics such as own values, women’s status in society and overcoming obstacles at work [50]. The Population Council’s Heshima Project in Kenya successfully used a similar approach [51]. While these training sessions have been implemented as in-service interventions for working midwives, it would be interesting to include and evaluate a similar module within the national curriculum for midwifery education.

Allowing birth companions during labour and delivery is highly recommended by WHO [52]. Our study confirmed the positive influence of birth companions for both the midwife and labouring women. Midwives believed birth companions can improve the reputation of the hospital by witnessing good care and have a positive influence on women’s wellbeing. Currently only women are allowed as birth companions in almost all health facilities in Mozambique, although the Ministry of Health would like to allow men on all maternity wards in the country in the long term [53]. Midwives referred to the private hospitals as providing a good example in this matter by allowing male partners. Unfortunately, the public health system does not seem to be prepared yet to allow men on the labour ward. The measures proposed in our study (training providers, preparing male partners during ANC and maintaining privacy for all women) will require investments in terms of infrastructure and human resources.

### Limitations

The setting of our study is limited to one hospital: a national referral hospital with very specific characteristics. This means that transferability beyond other similar settings is limited. However, while on the one hand we have findings that are very context specific (such as the interaction of midwives with patients that expect higher standards of care and prefer doctors), on the other hand we have findings that have been documented worldwide such as the vital role of supervision for tackling D&A [54] and importance of respect for midwives within the health system [41].

We lack evidence from the perspectives of doctors and health facility managers regarding their interactions with midwives and patients. Furthermore, the principle investigator of the study is a midwife herself, which may imply that the study only partially explores D&A from a limited perspective (that of midwives). Future research using triangulation of data coming from midwives, doctors, managers, women and their birth companions could reveal other perceptions about the essential aspects of respectful maternity care and ways to improve overall quality of care.

## Conclusion

In our study we explored two broad themes – midwives’ identity and occurrence of D&A – among midwives working in the national referral hospital of Mozambique. Results revealed some specific challenges for midwives working in a modernised capital in a LMIC. An increasing group of well-informed patients tended to show little respect or gratitude for midwives’ work because they prefer doctors as health care providers and expect a better service. In addition, midwives often faced disrespect by superiors within the health facility and felt treated unfairly compared to doctors. Their feeling of being disrespected contributed to D&A as an act of projecting their frustrations on patients. The involvement of birth companions together with supervision seemed to protect against D&A, and having a head midwife for supervisory support was mentioned as good practice.

Our study adds evidence to the relationship between midwives’ role and respect in society and the occurrence of D&A. It is important to recognize that midwives will need to be treated with more respect and dignity in Mozambique in order to guarantee the highest quality of care for mothers and their newborns. Only by guaranteeing availability of motivated and competent midwives equipped with essential physical resources can pregnant women and their newborns receive the highest standards of care as defined by the WHO framework for quality of care [8].

## Supplementary information

**Additional file 1.** Topic Guide

**Additional file 2.** A plot of the coding structure

**Additional file 3.** Code frequency per FGD

## Data Availability

The dataset of the current study is available from the corresponding author upon reasonable request.

## Abbreviations

ANC: Antenatal care
D&A: Disrespect and abuse
FGDs: Focus group discussions
HCM: Hospital central de Maputo
LMIC: Low and middle income countries
MMR: Maternal mortality ratio
MNH: Maternal and newborn health
MoH: Ministry of health
NGOs: Non-governmental organisations
RQDA: R qualitative data analysis
UEM: Universidade Eduardo Mondlane
WHO: World health organization
