# Supplementary material for: A qualitative study on midwives’ identity and perspectives on the occurrence of disrespect and abuse in Maputo city
Source: BMC Pregnancy Childbirth. 2020 Oct 19;20:629. doi: 10.1186/s12884-020-03320-0 (PMC7569757; doi:10.1186/s12884-020-03320-0)
Supplement: Supplementary file 1 — Additional file 1. Topic Guide [file 12884_2020_3320_MOESM1_ESM.docx]

DEMOGRAPHIC SURVEY

1. Sex: F / M

2. How old are you? __________

3. Are you married? yes / no

4. Do you have any children? yes / no

5. If yes, how many children? __________

6. What is your religion? __________

7. How many years of midwifery training have you had? _____ years.

8. How long have you been working as a midwife? __________

9. In which other health institutions did you work as a midwife and for how long?

Institution: Years:

Institution: Years:

Institution: Years:

Institution: Years:

10. At how many deliveries have you been the lead medical professional? _____

TOPIC GUIDE

1. Warm up

Please tell your first name, how long you have worked in HCM and if you worked in other places. Are you proud to be a midwife and what do you love most about your job?

1. Summary of what project includes and rationale of the current study

Remind them about the workshops and content regarding respectful maternity care. Discuss main results of prevalence study of D&A in Maputo Province by Galle et.al (2019).

1. Questions

What is respectful maternity care in their opinion?

Discuss specific aspects of RMC:

**Lack of confidentiality/privacy**: What are the reasons for this? Is it a prevalent problem because of infrastructure?

**Being left alone**: Why do women feel they are being left alone too often? Is the work pressure to high?

**Being shouted at/scolded**: Why do some providers sometimes shout? Is this related to work pressure/patients behavior? When is it justified to shout/scold?

**Being given a treatment without permission**: Do providers always ask permission before injections/examinations? Is this always possible? Why not? Does counselling about given treatment always happen? Why (not)? Does the patient have any say in treatment/medications she receives (eg induction, use of oxytocin, birth position)?

Women have the **right to choose their own birth companion but men are not allowed**: what is the reason? If privacy can be guaranteed are there any problems? Would you be in favor of allowing men? Why is this (not) a good idea?

**Profession of midwives:** Do you feel respected in the society/hospital/team? Work pressure? Salary? Hierarchy?

Please describe how your training has covered “patients’ rights”, if at all. What do ‘patients’ rights’ mean to you?

1. Closure

We want to stress again that all information is confidential and that information only will be used to improve care in general. Thank you!
