# Supplementary figures and images for: A qualitative study on midwives’ identity and perspectives on the occurrence of disrespect and abuse in Maputo city
Source: BMC Pregnancy Childbirth. 2020 Oct 19;20:629. doi: 10.1186/s12884-020-03320-0 (PMC7569757; doi:10.1186/s12884-020-03320-0)

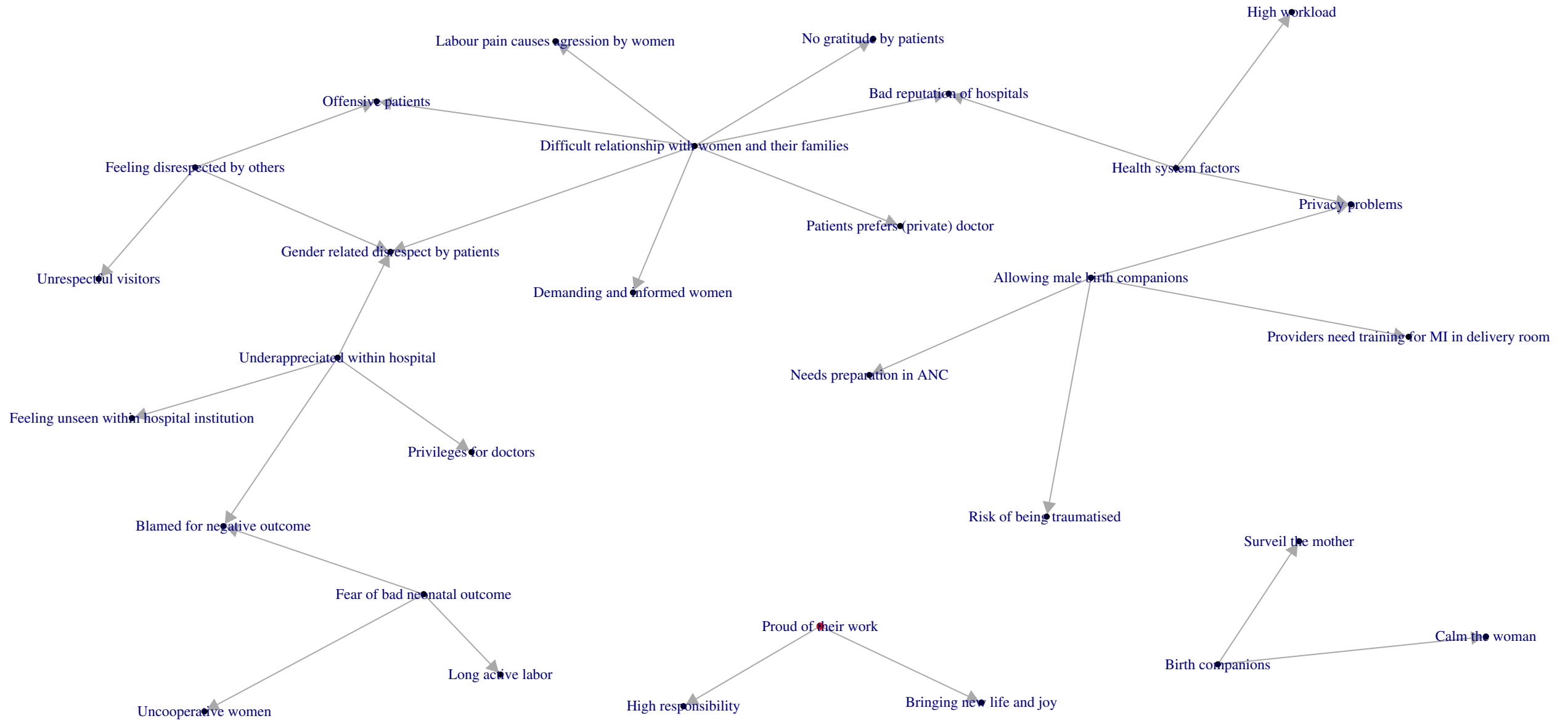

Supplement: Supplementary file 2 — Additional file 2. A plot of the coding structure [file 12884_2020_3320_MOESM2_ESM.pdf]

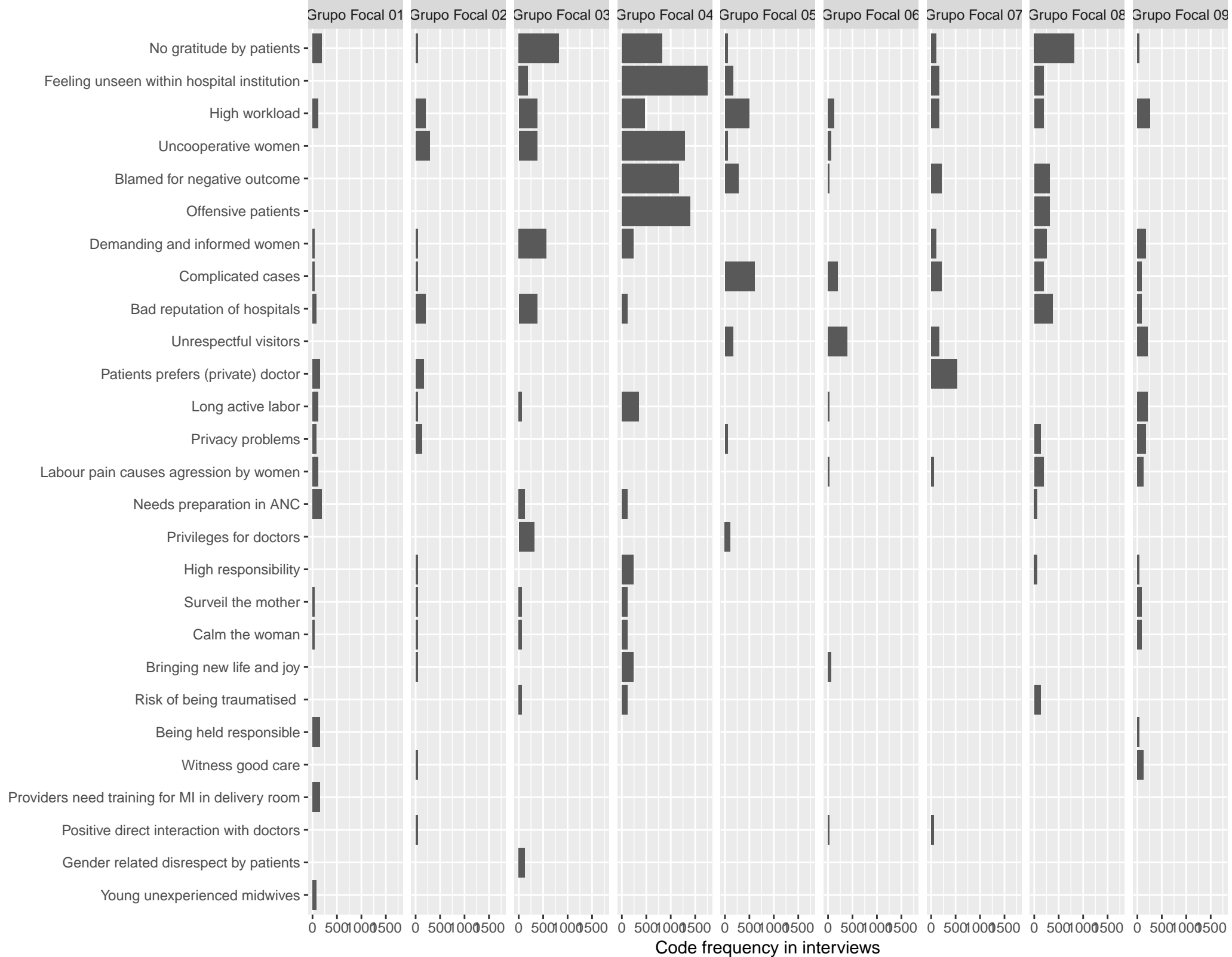

Supplement: Supplementary file 3 — Additional file 3. Code frequency per FGD [file 12884_2020_3320_MOESM3_ESM.pdf]
